# Generation of HIV-resistant cells with a single-domain antibody: implications for HIV-1 gene therapy

**DOI:** 10.1038/s41423-020-00627-y

**Published:** 2021-01-18

**Authors:** Hongliang Jin, Xiaoran Tang, Li Li, Yue Chen, Yuanmei Zhu, Huihui Chong, Yuxian He

**Affiliations:** 1 NHC Key Laboratory of Systems Biology of Pathogens, Institute of Pathogen Biology, Chinese Academy of Medical Sciences and Peking Union Medical College, Beijing, China; 2 Center for AIDS Research, Chinese Academy of Medical Sciences and Peking Union Medical College, Beijing, China

**Keywords:** HIV-1, gene therapy, resistant cell, single-domain antibody, glycosylphosphatidylinositol, Immunosuppression, Mechanisms of disease

## Abstract

The cure or functional cure of the “Berlin patient” and “London patient” indicates that infusion of HIV-resistant cells could be a viable treatment strategy. Very recently, we genetically linked a short-peptide fusion inhibitor with a glycosylphosphatidylinositol (GPI) attachment signal, rendering modified cells fully resistant to HIV infection. In this study, GPI-anchored m36.4, a single-domain antibody (nanobody) targeting the coreceptor-binding site of gp120, was constructed with a lentiviral vector. We verified that m36.4 was efficiently expressed on the plasma membrane of transduced TZM-bl cells and targeted lipid raft sites without affecting the expression of HIV receptors (CD4, CCR5, and CXCR4). Significantly, TZM-bl cells expressing GPI-m36.4 were highly resistant to infection with divergent HIV-1 subtypes and potently blocked HIV-1 envelope-mediated cell-cell fusion and cell-cell viral transmission. Furthermore, we showed that GPI-m36.4-modified human CEMss-CCR5 cells were nonpermissive to both CCR5- and CXCR4-tropic HIV-1 isolates and displayed a strong survival advantage over unmodified cells. It was found that GPI-m36.4 could also impair HIV-1 Env processing and viral infectivity in transduced cells, underlying a multifaceted mechanism of antiviral action. In conclusion, our studies characterize m36.4 as a powerful nanobody that can generate HIV-resistant cells, offering a novel gene therapy approach that can be used alone or in combination.

## Introduction

Highly active antiretroviral therapy (HAART), which employs multiple drugs in combination, can efficiently suppress HIV-1 replication, which has dramatically reduced the morbidity and mortality associated with AIDS and the risk of HIV-1 transmission. However, HAART cannot eradicate latent virus, necessitating lifelong treatment that often causes cumulative toxicities and drug resistance^1,2^. Several vaccines designed to elicit broadly neutralizing antibodies (bNAbs) against HIV-1 have been developed, but they exhibit poor or no protective efficacy^3–5^. Recent studies suggest that passive infusion of bNAbs can provide therapeutic effects in HIV-1-infected individuals^6–9^; however, the concentration of infused bNAbs wanes over time, and repeated administrations are required to maintain long-term viral suppression. Similar to HAART, soluble bNAbs are not curative. Thus, the development of sterilizing or functional curative strategies has become a priority in the fight against HIV/AIDS.

Groundbreaking progress was made with the achievement of a cure or functional cure in the “Berlin patient” and “London patient”, who were transplanted with allogeneic hematopoietic stem cells (HSCs) harboring a naturally occurring mutation in the coreceptor CCR5 (CCR5Δ32) for the treatment of hematological malignancies^10,11^. In contrast, only transient HIV-1 remission was observed in two “Boston patients” and one “Minnesota patient” who received allogeneic transplantation of HSCs without CCR5Δ32^12,13^. These discoveries indicate that the impact of CCR5Δ32 is critical in viral suppression or eradication and that infusion of HIV-resistant cells would be a viable treatment strategy for effecting an HIV-1 cure. Thus, considerable effort is being exerted to engineer CCR5 mutations into autologous cells using gene-editing technologies; however, such an approach has so far achieved only very limited therapeutic efficacy in patients^14–16^. Very recently, Xu et al. reported successful allogeneic transplantation and long-term engraftment of HSCs with CCR5 ablation by a CRISPR/Cas9-based gene editing system in a patient with HIV-1 infection and acute lymphoblastic leukemia, which demonstrated safety but showed no therapeutic effect in terms of viral replication^14^. Therefore, it is imperative to develop safer and more effective gene therapy-based methods utilizing a patient’s own cells to simultaneously protect against infection and promote immune reconstruction. Conceivably, HIV-1 entry inhibitors, such as bNAbs and fusion inhibitor peptides, would provide advantages in generating HIV-resistant cells because they can block the gateway of viral entry.

The entry of HIV-1 into target cells is mediated by viral envelope (Env) glycoproteins: the surface subunit gp120 is responsible for binding with the primary cell receptor CD4 and a coreceptor (CCR5 or CXCR4), which triggers substantial conformational changes in the viral Env complex, while the transmembrane subunit gp41 is involved in virus-cell fusion by inserting its fusion peptide into the cell membrane and then folding a six-helix bundle (6-HB) structure between its N- and C-terminal heptad repeat regions^17^. Currently, there are three clinically approved drugs targeting the HIV-1 entry step: the first is a gp41-derived peptide fusion inhibitor (Enfuvirtide, T-20)^18^, the second is a small-molecule CCR5 antagonist (Maraviroc) that is being used to treat CCR5-tropic HIV-1 infection^19^, and in 2018, a humanized anti-CD4 antibody (ibalizumab) was licensed for clinical use^20^. In contrast to other drugs that act after infection, entry inhibitors intercept HIV-1 before it invades target cells. In the past decade, we have focused on developing peptide-based HIV-1 fusion inhibitors, reporting a group of peptides and lipopeptides with extremely potent antiviral activity^21–32^; we have also focused on identifying and characterizing human neutralizing antibodies from HIV-1-infected individuals, such as A16^33^, Y498^34^, and m36.4. Previous studies have demonstrated that m36.4 is a single-domain antibody (nanobody) targeting the receptor CD4-induced (CD4i) epitope that overlaps with the coreceptor-binding site on gp120^35–37^. To develop a gene therapy-based strategy for HIV-1 functional cure, we recently generated HIV-resistant cells by genetically expressing a short-peptide fusion inhibitor on the plasma membrane of target cells through the glycosylphosphatidylinositol (GPI) attachment signal of decay accelerating factor (DAF)^38^. In this study, we constructed fusion genes encoding GPI-anchored m36.4 (GPI-m36.4) or a GPI-anchored single-chain variable fragment (scFv) of the anti-avian influenza virus hemagglutinin (HA) control antibody FluIgG03^39^. We verified that both GPI-m36.4 and GPI-FluIgG03 were efficiently expressed within the plasma membrane lipid raft sites of transduced TZM-bl cells without interfering with the expression of HIV receptors; however, GPI-m36.4 but not GPI-FluIgG03 specifically rendered target cells highly resistant to divergent HIV-1 infections, viral Env-mediated cell–cell fusion, and cell-associated virion-mediated cell–cell transmission. More importantly, GPI-m36.4-modified human CD4+ cells (CEMss-CCR5) were exceptionally resistant to both CCR5- and CXCR4-tropic HIV-1 isolates and had a robust selective survival advantage over unmodified cells following HIV-1 infection. The disruptive effects of GPI-m36.4 on HIV-1 Env processing and viral infectivity were further characterized, verifying a multifaceted mode of antiviral action. Therefore, the present studies have provided a potential HIV-1 gene therapy approach that can be used alone or in combination with other strategies.

## Results

### Construction of lentiviral vectors for cell-surface expression of m36.4 through a GPI anchor

**Fig. 1:**
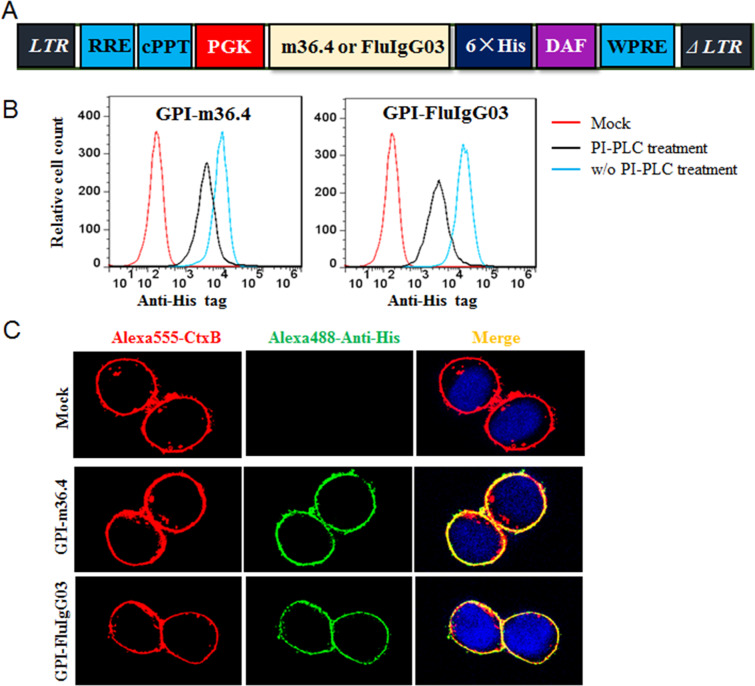
Cell-surface expression of GPI-anchored antibodies in transduced TZM-bl cells. **A** Diagram of the lentiviral transfer vector expressing GPI-anchored antibodies. The encoding sequence of m36.4 or FluIgG03 scFv was genetically linked with sequences encoding a His tag and the GPI attachment signal of DAF. RRE, Rev response element; cPPT, central polypurine track; WPRE, woodchuck hepatitis virus posttranscriptional regulatory element. **B** Expression of GPI-m36.4 or GPI-FluIgG03 on the surface of transduced TZM-bl cells with or without PI-PLC treatment, which was detected by an anti-His tag antibody and analyzed by FACS analysis. **C** Localization of GPI-m36.4 or GPI-FluIgG03 in transduced TZM-bl cells with confocal analysis. Alexa555-CtxB, cells stained with an Alexa Fluor 555-conjugated cholera toxin B subunit; Alexa488-Anti-His: cells stained with a mouse anti-His tag antibody followed by an Alexa Fluor 488-conjugated goat anti-mouse IgG antibody

To develop HIV-resistant cells as a gene therapy approach, fusion genes encoding the single-domain nanobody m36.4 or the scFv format of the control antibody FluIgG03 were constructed for cell-surface expression through a GPI anchoring approach. As illustrated in Fig. 1A, the m36.4- or FluIgG03 scFv-encoding sequence was genetically fused with sequences encoding a His-tag and the GPI attachment signal of DAF and then inserted into a self-inactivating lentiviral vector (pRRLsin.PPT.hPGK.WPRE). The recombinant lentiviruses were produced by cotransfecting HEK293T cells with the transfer vector, a packaging plasmid encoding Gag/Pol/Rev, and a plasmid encoding vesicular stomatitis virus G glycoprotein (VSV-G). TZM-bl cells were then transduced with the recombinant lentiviruses and sorted by fluorescence-activated cell sorting (FACS) to isolate a cell population in which close to 100% of the cells expressed GPI-anchored m36.4 (GPI-m36.4) or FluIgG03 scFv (GPI-FluIgG03). To verify whether the fusion genes were expressed on the cell surface through the GPI anchor, transduced TZM-bl cells were treated with phosphatidylinositol-specific phospholipase C (PI-PLC) and then stained with an anti-His tag antibody, followed by FACS analysis. As shown in Fig. 1B, both of the transgenes were efficiently expressed on the surface of transduced cells, but their expression greatly decreased after PI-PLC treatment, confirming that the m36.4 and FluIg03 antibodies were tethered to the cell membrane through a GPI anchor.

### GPI-m36.4 and GPI-FluIg03 are primarily directed to lipid rafts and do not affect the expression of HIV receptors

GPI anchoring in nature is a typical posttranslational modification process, and many GPI-linked proteins are directed to lipid rafts, which are specialized dynamic microdomains in the cell plasma membrane. Coincidently, lipid raft sites also host the HIV-1 receptor CD4 and serve as gateways for HIV entry and budding^40,41^. We previously verified that the GPI-anchored fusion inhibitor peptide 2P23 was mainly located within lipid rafts^38^. Herein, we characterized whether newly designed GPI-m36.4 and GPI-FluIg03 resided in lipid rafts. To this end, GPI-m36.4- and GPI-FluIg03-transduced TZM-bl cells were costained with (i) a mouse anti-His tag antibody, followed by an Alexa Fluor 488-conjugated goat anti-mouse IgG antibody, (ii) Alexa Fluor 555-conjugated cholera toxin subunit B (CtxB), which can interact with the lipid raft marker GM1, and (iii) 4′,6-diamidino-2-phenylindole (DAPI). As analyzed by confocal microscopy (Fig. 1C), both GPI-m36.4 and GPI-FluIg03 colocalized with GM1 on the cell surface, indicating that they were indeed localized in the lipid raft sites within the plasma membrane.

**Fig. 2:**
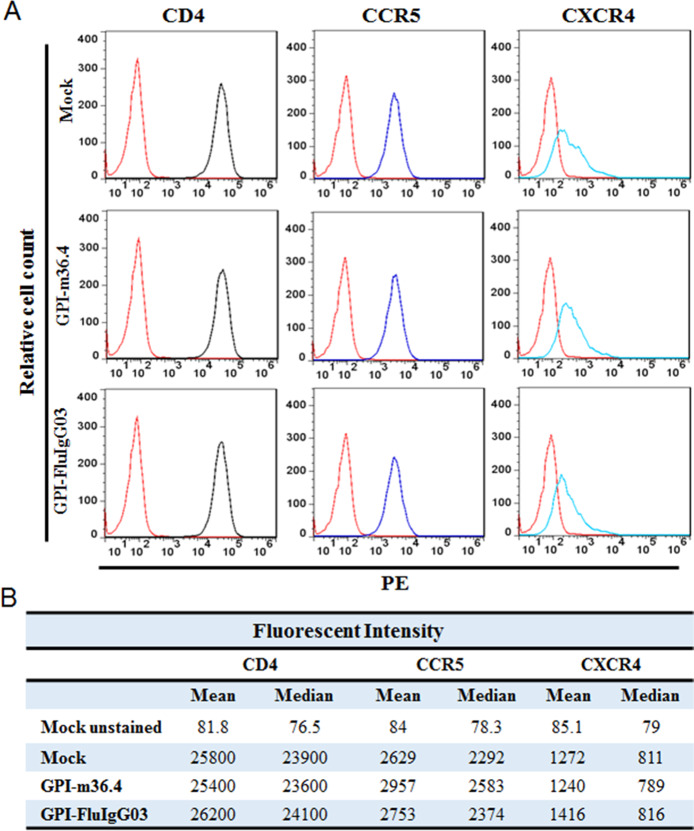
Effects of GPI-anchored antibodies on the expression of HIV receptors. The expression of CD4, CCR5 or CXCR4 on the surface of TZM-bl cells transduced with a GPI-anchored scFv or m36.4 was detected with a PE-conjugated anti-human CD4 (black), CCR5 (blue), or CXCR4 (cyan) antibody and analyzed by FACS analysis. The expression levels were assessed by measuring the relative cell counts (**A**) and the fluorescence intensity (**B**)

To determine whether the cell-surface anchoring and expression of a domain antibody or scFv interfered with the expression of the primary receptor CD4 and coreceptors CCR5 and CXCR4, we stained mock-, GPI-m36.4-, and GPI-FluIg03-transduced TZM-bl cells with a PE-conjugated antihuman CD4, CCR5, or CXCR4 antibody and analyzed the cells by FACS analysis. As shown in Fig. 2, the relative cell counts and fluorescence intensities of the TZM-bl cells transduced with GPI-m36.4 or GPI-FluIg03 were comparable to those of the parental TZM-bl cells, suggesting that the expression of GPI-m36.4 and GPI-FluIg03 had no appreciable effect on the cell-surface expression of the HIV-1 receptor and coreceptors. Furthermore, we did not observe any harmful effects of the constructs on the viability and growth of the transduced cells (data not shown). Moreover, the vector copy number (VCN) in the transduced cells was analyzed by real-time quantitative PCR analysis (qPCR), revealing an average of 5.7 copies per cell (Supplementary Fig. S1).

### GPI-m36.4-modified cells are highly resistant to divergent HIV-1 infections

We next sought to characterize the resistance profiles of TZM-bl cells transduced with GPI-m36.4. First, a panel of eight replication-competent HIV-1 isolates with different tropisms was used to infect transduced cells. As shown in Table 1, control antibody GPI-FluIgG03-transduced TZM-bl cells were highly susceptible to infection with the eight HIV-1 isolates including five CCR5-tropic viruses (JR-CSF, RHPA.c/2635, THRO.c/2626, CH077.t/2627, and MJ4), two CXCR4-tropic viruses (NL4-3 and LAI.2), and one dual-tropic virus (89.6). Significantly, the TZM-bl cells expressing GPI-m36.4 were fully resistant to all HIV-1 isolates tested. To verify the anti-HIV breadth and potency of GPI-m36.4 in TZM-bl cells, we further applied a “global panel” of HIV-1 pseudoviruses, which included 12 diverse subtypes of viral Envs representing the global AIDS epidemic^42^. As determined by a single-cycle infection assay, transduced cells displayed very high levels of resistance to divergent HIV-1 subtypes, except for the pseudoviruses 398-F1_F6_20, X1632-S2-B10, CNE8, and CNE55, which infected the cells with average infection rates of 6%, 3%, 3%, and 1%, respectively (Table 1). In regard to virus control, GPI-m36.4 and GPI-FluIgG03 did not confer resistance to VSV-G, indicating the antiviral specificity of GPI-m36.4 in transduced cells. To verify the functionality of GPI-mediated inhibitor anchoring, we also constructed lentiviral vectors that expressed secretory m36.4 (Sec-m36.4) and FluIgG03 scFv (Sec-FluIgG03), and their antiviral activities were examined with the same panels of replication-competent and Env-pseudotyped viruses. As shown in Table 1, the TZM-bl cells transduced with Sec-m36.4 could not effectively block infection with the HIV-1 isolates or VSV-G control. A Western blot assay with an anti-His tag antibody found that Sec-m36.4 and Sec-FluIgG03 were detected only in the highly concentrated cell culture supernatants, not in the cell lysates (Supplementary Fig. S2). Moreover, it was expected that the amount of Sec-m36.4 in the supernatants of infected cultures was not sufficient to inhibit the viruses, consistent with previous findings generated with similar protocols^43,44^.

**Table 1:** Inhibitory activity of GPI-anchored m36.4 in TZM-b1 cells against divergent HIV-1 subtypes

HIV-1	Subtype	Tropism	Mean % infection ± SD			
			GPI-FluIgG03	GPI-m36.4	Sec-FluIgG03	Sec-m36.4
Replication-competent virus						
JR-CSF	B	CCR5	96 ± 3	0	128 ± 8	65 ± 5
RHPA.c/2635	B	CCR5	106 ± 7	0	120 ± 10	61 ± 4
THRO.c/2626	B	CCR5	92 ± 2	0	116 ± 4	53 ± 6
CH077.t/2627	B	CCR5	89 ± 3	0	108 ± 1	69 ± 4
NL4-3	B	CXCR4	85 ± 2	0	114 ± 5	43 ± 5
LAI.2	B	CXCR4	82 ± 3	0	120 ± 4	56 ± 5
89.6	B	R5X4	90 ± 4	0	118 ± 6	59 ± 4
MJ4	C	CCR5	98 ± 5	0	112 ± 8	74 ± 3
"Global Panel" pseudovirus						
398-F1_F6_20	A	CCR5	141 ± 4	6 ± 1	102 ± 6	72 ± 4
TRO.11	B	CCR5	104 ± 1	0	113 ± 8	89 ± 5
X2278_C2_B6	B	CCR5	136 ± 8	0	106 ± 2	80 ± 3
CE1176_A3	C	CCR5	115 ± 1	0	101 ± 3	84 ± 4
CE703010217_B6	C	CCR5	112 ± 2	0	88 ± 3	75 ± 5
HIV_25710-2.43	C	CCR5	117 ± 2	0	106 ± 6	80 ± 4
X1632-S2-B10	G	CCR5	108 ± 0	3 ± 1	106 ± 2	82 ± 3
246_F3_C10_2	A/C	CCR5	113 ± 2	0	105 ± 4	87 ± 4
CNE8	A/E	CCR5	139 ± 2	3	107 ± 5	93 ± 2
CNE55	A/E	CCR5	126 ± 2	1	103 ± 6	90 ± 6
CH119.10	B/C	CCR5	112 ± 2	0	111 ± 8	98 ± 7
BJOX002000.03	B/C	CCR5	106 ± 2	0	103 ± 5	93 ± 5
VSV-G	NA	NA	97 ± 1	103 ± 2	102 ± 7	111 ± 7

*The assay was performed in triplicate and repeated three times. Data are expressed as the means ± SD

### GPI-m36.4-modified cells are highly resistant to HIV-1 Env-mediated cell–cell fusion

We previously applied a DSP-based cell-cell fusion assay to assess the antiviral activity of various HIV-1 fusion inhibitors, wherein 293FT cells expressing CCR5/CXCR4/DSP_8-11_ were used as a target (designated 293FT_Target_ cells). In this study, we investigated the resistance profiles of m36.4-modified cells for HIV-1 Env-mediated cell-cell fusion. Thus, 293FT_Target_ cells were transduced with GPI-m36.4 or GPI-FluIgG03 for characterization. Similarly, FACS analysis was first performed to examine the cell-surface expression of the transgenes and their effects on the expression of CD4, CCR5, and CXCR4. As anticipated, GPI-m36.4 and GPI-FluIgG03 were efficiently expressed on the surface of 293FT_Target_ cells and did not significantly affect the expression levels of the receptor and coreceptors, as judged by fluorescence intensities (Supplementary Fig. S3). Subsequently, the DSP-based fusion assay was conducted to quantitate the inhibitory activities of GPI-m36.4 and GPI-FluIgG03 in viral Env-mediated cell–cell fusion. Similar to their inhibitory activities against both wild-type HIV-1 and pseudotyped HIV-1 infections, the transduction of GPI-FluIgG03 did not impart cell resistance, whereas GPI-m36.4-transduced cells were highly resistant to cell fusion mediated by HIV-1 Env proteins with divergent subtypes and phenotypes (Table 2).

**Table 2:** Inhibitory activity of GPI-m36.4 on HIV-1 Env-mediated cell–cell fusion

HIV-1 Env	Subtype	Tropism	Mean % cell fusion ± SD	
			GPI-FluIgG03	GPI-m36.4
398-F1_F6_20	A	CCR5	108 ± 4	16 ± 5
92RW020	A	CCR5	113 ± 2	0
92UG037.8	A	CCR5	106 ± 4	0
NL4-3	B	CXCR4	105 ± 4	0
SF162	B	CCR5	105 ± 4	0
R3A	B	CCR5	98 ± 3	0
CNE11	B'	R5X4	110 ± 10	0
ZM53M.PB12	C	CCR5	108 ± 7	0
HIV_25710-2.43	C	CCR5	108 ± 5	0
GX11.13	A/E	CCR5	100 ± 6	5 ± 1
CNE8	A/E	CCR5	107 ± 8	11 ± 3
CNE55	A/E	CCR5	107 ± 10	10 ± 3
CH70.1	B/C	R5X4	100 ± 2	0
CH119.10	B/C	CCR5	106 ± 5	0
BJOX002000.03	B/C	CCR5	110 ± 4	0

*The assay was performed in triplicate and repeated three times. Data are expressed as the means ± SD

### GPI-m36.4-modified cells are fully resistant to cell-cell HIV-1 transmission

**Fig. 3:**
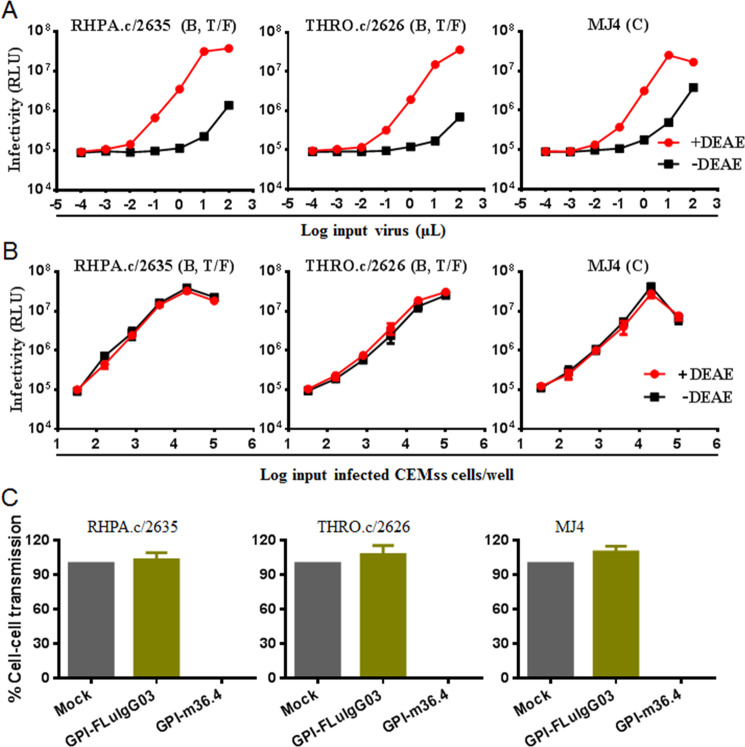
Inhibitory activity of GPI-anchored antibodies during cell–cell HIV-1 transmission. **A** Infection with the replication-competent CCR5-tropic HIV-1 isolates RHPA.c/2635 (subtype B), THRO.c/2626 (subtype B), and MJ4 (subtype C) in TZM-bl cells depends on the presence of DEAE-dextran when the isolates are in the form of cell-free viruses. RLU, relative luciferase units; T/F, transmitted/founder virus. The assay was performed in triplicate and repeated two times, and representative data are shown. **B** Infection with cell-associated RHPA.c/2635, HRO.c/2626, and MJ4 in TZM-bl cells was independent of DEAE-dextran during cell-cell transmission. The assay was performed in triplicate and repeated two times, and representative data are shown. **C** Inhibition of cell-cell HIV-1 transmission by GPI-anchored antibodies. TZM-bl cells expressing GPI-m36.4 or GPI-FluIgG03 were used as targets and cocultured with CEMss-CCR5 donor cells that were preinfected with one of the three viruses. % cell-cell transmission was monitored by quantifying the production of the luciferase reporter in the TZM-bl cells. Error bars represent the means ± standard deviations (SD) of three independent experiments with triplicate samples

Cell-cell transmission is also considered a crucial pathway of HIV-1 spread in infected individuals; however, recent studies suggest that many bNAbs have a relatively low capacity to block cell-cell transmission mediated by cell-associated HIV-1 virions^45–47^. Therefore, we thought it would be interesting to characterize the neutralizing activity of membrane-anchored anti-HIV single-domain antibodies. To this aim, we adopted an experimental protocol that was recently used to evaluate the GPI-anchored fusion inhibitor 2P23^38^, the rationale of which was based on the observation that coculturing CCR5-tropic HIV-infected CEMss-CCR5 cells (donor) with TZM-bl cells (target) resulted in rapid and efficient infection by the virus through a cell-cell pathway. Consistent with our previous results^38^, the infectivity of three cell-free viruses, including two subtype B transmitted/founder viruses (RHPA.c/2635, THRO.c/2626) and one subtype C virus (MJ4), in TZM-bl cells sharply decreased in the absence of a polycationic DEAE-dextran supplement (Fig. 3A), whereas the cell-cell transmission of the cell-associated viruses (CEMss-CCR5^HIV+^) was independent of DEAE-dextran (Fig. 3B). Accordingly, TZM-bl target cells were transduced with GPI-m36.4 or GPI-FluIgG03 and cocultured with CEMss-CCR5^HIV+^ cells without the addition of DEAE-dextran. As shown in Fig. 3C, while TZM-bl cells expressing GPI-FluIgG03 were effectively infected by the three tested viruses from CEMss-CCR5^HIV+^ donor cells, the TZM-bl cells expressing GPI-m36.4 were fully resistant to transmission.

### GPI-m36.4 renders human CD4^+^ T cells highly resistant to divergent HIV-1 strains

**Fig. 4:**
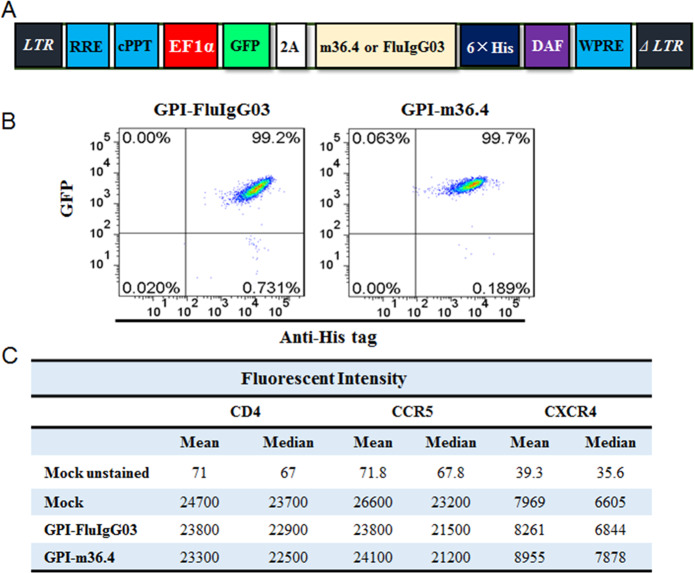
Expression of GPI-anchored antibodies fused with GFP in transduced human CD4+ T cells and their effects on CD4, CCR5, and CXCR4. **A** Diagram of the lentiviral transfer vectors expressing GPI-m36 or GPI-FluIgG03 linked to GFP-encoding sequences via a 2A signal. **B** FACS analysis of the cell-surface expression of GPI-m36.4 or GPI-FluIgG03 and GFP in transduced TZM-bl cells. **C** Expression of CD4, CCR5, or CXCR4 on the surface of transduced CEMss-CCR5 cells, as evaluated by measuring the fluorescence intensity

The results above demonstrated that GPI-m36.4 could endow target cells with potent resistance to divergent HIV-1 isolates. We next focused on characterizing whether GPI-m36.4 is capable of protecting human CD4^+^ T cells from HIV-1 infection. To facilitate the monitoring of transduced cells, transgenes were designed to express GPI-anchored m36.4 or FluIgG03 genetically linked to green fluorescent protein (GFP) through an internal 2A protein splicing signal in the lentiviral transfer vector pRRLsin-18.PPT.EF1α.WPRE (Fig. 4A). The recombinant lentiviruses were packaged and transduced into human CEMss-CCR5 cells. As detected with GFP and an anti-His antibody, GPI-m36.4/GFP and GPI-FluIgG03/GFP were efficiently expressed on the cell surface of CEMss-CCR5 cells (Fig. 4B). Consistently, the expression of CD4, CCR5, and CXCR4 in the transduced cells was not significantly affected by the transgenes (Fig. 4C). In the transduced CEMss-CCR5 cells, an average VCN of 1.1 copies per cell was detected by qPCR (Supplementary Fig. S1).

**Fig. 5:**
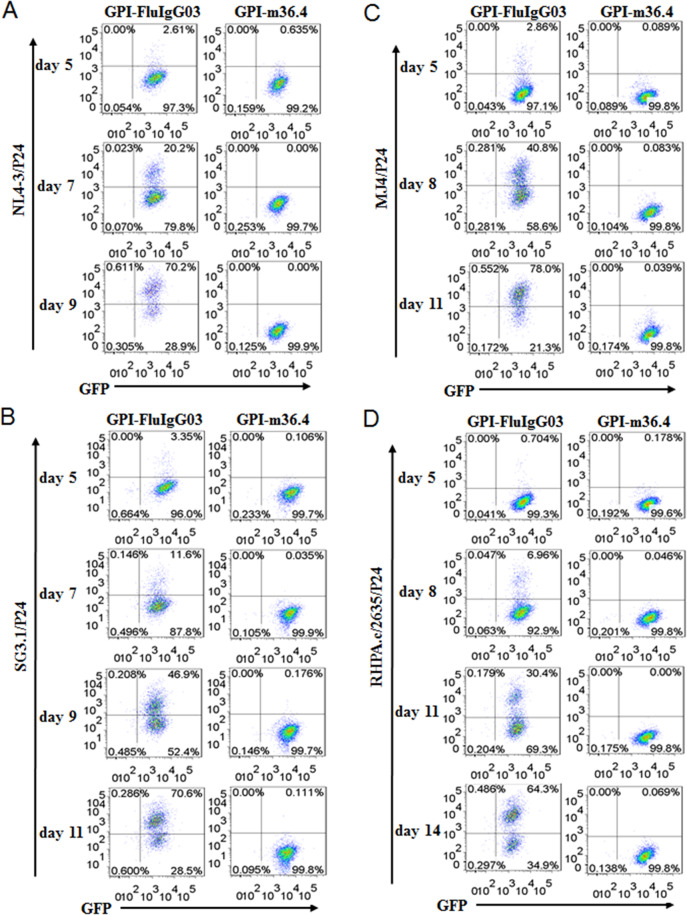
Inhibitory activity of GPI-anchored antibodies in transduced human CD4+ T cells during HIV-1 infection. CEMss-CCR5 cells transduced with GPI-m36.4/GFP or GPI-FluIgG03/GFP were infected with 1,000 TCID_50_ of NL4-3 (**A**), SG3.1 (**B**), MJ4 (**C**), or RHPA.c/2635 (**D**), and intracellular HIV-1 P24 Gag and GFP expression was monitored over time by flow cytometry. Data from an experiment representative of three independent experiments are shown

To evaluate the inhibitory activities of GPI-m36.4 and GPI-FluIgG03, transduced CEMss-CCR5 cells were first infected with two CXCR4-tropic strains (NL4-3 and SG3.1) and then cultured in complete DMEM for 9 or 11 days. The infected cells were monitored over time for intracellular HIV-1 P24-Gag and GFP expression by flow cytometry. As controls, GPI-FluIgG03/GFP-transduced CEMss-CCR5 cells showed 2.61%, 20.2%, and 70.2% P24-Gag and GFP double-positive cells after NL4-3 inoculation for 5, 7, and 9 days, respectively, and 3.35%, 11.6%, 46.9%, and 70.6% P24-Gag and GFP double-positive cells after SG3.1 inoculation for 5, 7, 9, and 11 days, respectively (Fig. 5A, B, left panels), indicating increased infection over time; in sharp contrast, no or very minor (<0.64%) proportions of P24-Gag and GFP double-positive cells were observed in the CEMss-CCR5 cell population expressing GPI-m36.4/GFP (Fig. 5A, B, right panels) at 9 or 11 days.

Next, we examined the inhibitory activities of GPI-m36.4 and GPI-FluIgG03 in transduced CEMss-CCR5 cells against two CCR5-tropic strains (MJ4 and RHPA.c/2635). Similarly, the GPI-FluIgG03/GFP-transduced CEMss-CCR5 cells were efficiently infected by MJ4 or RHPA.c/2635 over time (Fig. 5C, D, left panels); however, there were no or extremely few P24-Gag and GFP double-positive cells seen in the CEMss-CCR5 cells expressing GPI-m36.4/GFP (Fig. 5C, D, right panels) at 11 or 14 days. Taken together, these data indicate that GPI-anchored m36.4 can render CD4^+^ T cells extremely resistant to both CCR5- and CXCR4-tropic HIV-1 isolates. For clarity, the results are also shown in Supplementary Fig. S4.

### GPI-m36.4-modified CEMss-CCR5 cells possess a selective survival advantage following HIV-1 challenge

**Fig. 6:**
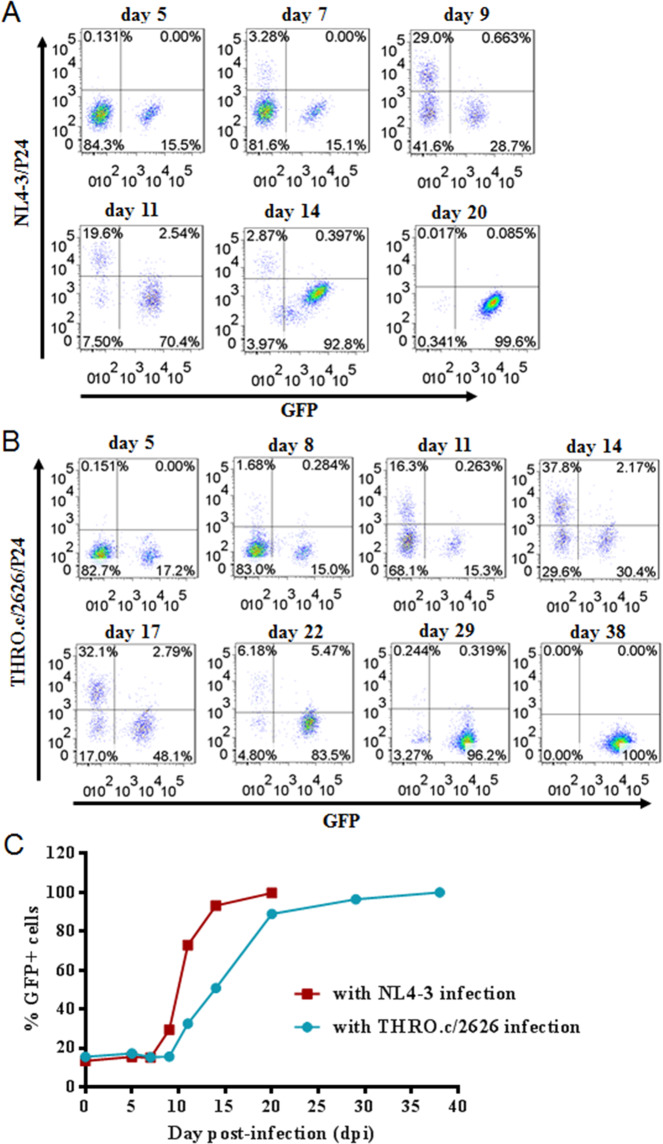
Selective survival of human CD4+ T cells expressing GPI-m36.4 during divergent HIV-1 infections. CEMss-CCR5 cells were transduced with GPI-m36.4/GFP and mixed with untransduced cells at a proportion of approximately 17% GFP-positive cells. The mixed population was challenged with 1,000 TCID_50_ of CXCR4-tropic NL4-3 (**A**) or CCR5-tropic THRO.c/2626 (**B**), and the proportion of transgene-expressing cells was monitored over time by flow cytometry. **C** Survival curves of transduced CEMss-CCR5 cells. Data from an experiment representative of three independent experiments are shown

We were interested in determining whether GPI-anchored m36.4 can produce selective survival and expansion advantages in transduced human CD4^+^ T cells compared to unmodified cells during HIV-1 infection, which is a key point as a potential gene therapy approach. To study this, ~15–20% GPI-m36.4/GFP-expressing CEMss-CCR5 cells were mixed with untransduced cells prior to HIV-1 challenge. Following viral infection with CXCR4-tropic NL4-3 (Fig. 6A) or CCR5-tropic THRO.c/2626 (Fig. 6B), the transduced cells exhibited gradually increased percentages of GFP-positive (GFP+) cells over time. In the NL4-3-infected culture, 99.6% of the cells expressed GPI-m36.4/GFP at day 20; in the THRO.c/2626-infected culture, 100% of the cells expressed GPI-m36.4/GFP at day 38 (Fig. 6C). In the absence of viruses, the proportions of GFP+ cells in the mixed populations were relatively stable (data not shown). In the control group, GPI-FluIgG03/GFP-expressing CEMss-CCR5 cells did not show resistance or selective survival and died over time (Supplementary Fig. S5). These results indicate that GPI-m36.4 is capable of conferring robust selective survival and expansion advantages to human CD4^+^ T cells following HIV-1 infection.

### GPI-m36.4 can inhibit the infectivity of progeny HIV-1 viruses and interfere with the processing of the Env glycoprotein

**Fig. 7:**
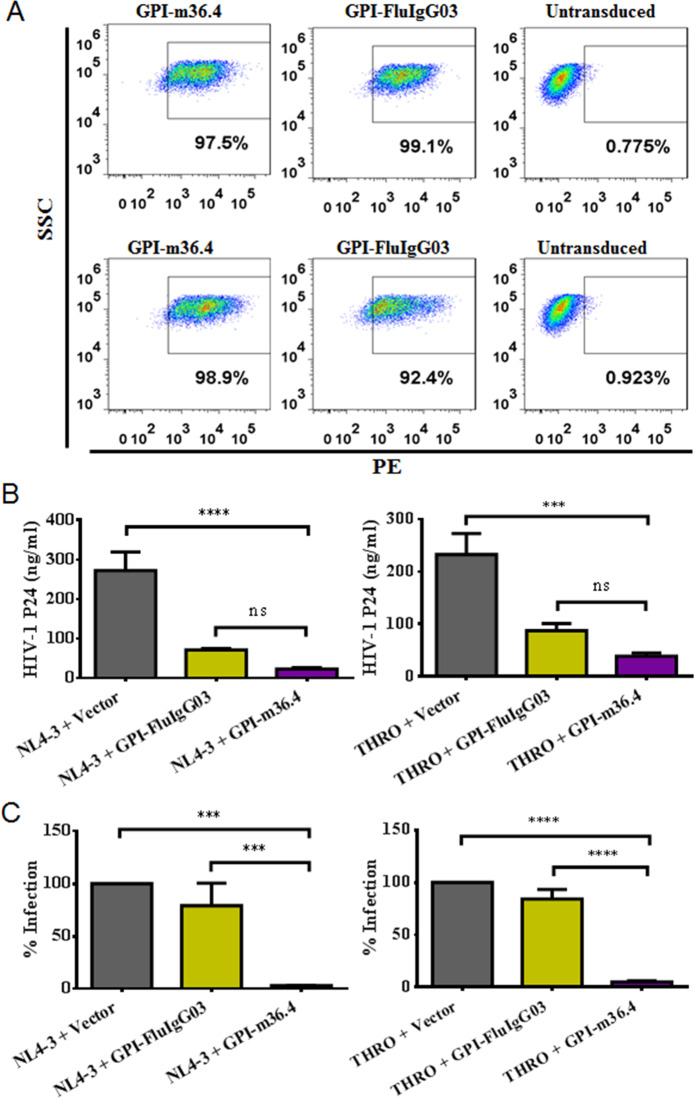
Cell-surface expression of GPI-anchored antibodies and their effects on HIV-1 release and viral infectivity. **A** Expression levels of GPI-anchored antibodies after cotransfection with an HIV-1 proviral clone. HKE293T cells were cotransfected with GPI-m36.4 or GPI-FluIgG03 and a proviral clone of HIV-1 NL4-3 (upper panel) or THRO.c/2626 (lower panel). The expression levels of GPI-m36.4 and GPI-FluIgG03 were detected by FACS analysis with an anti-His tag antibody. SSC, side scatter. **B** Effects of GPI-anchored antibodies on HIV-1 virion release. HEK293T cells were cotransfected with GPI-FluIgG03 or GPI-m36.4 and the HIV-1 provirus NL4-3 (left) or THRO.c/2626 (right). The amounts of the P24 antigen in cell culture supernatants were measured by ELISA. **C** Effects of GPI-anchored antibodies on the infectivity of progeny HIV-1 virions. To measure the infectivity of progeny NL4-3 (left) or THRO.c/2626 virions, cell culture supernatants containing equal amounts of the P24 antigen were used to infect TZM-bl cells, and luciferase activity in cell lysates was measured. The infectivity of viruses produced from empty vector-cotransfected cells was set at 100%, and the relative infection levels of HIV-1 produced from cells cotransfected with GPI-m36.4 or GPI-FluIgG03 were calculated accordingly. The data shown were derived from three independent experiments, and error bars indicate standard deviations. Statistical comparisons were conducted by ANOVA (***P < 0.001; ****P < 0.0001; ns, not significant)

Previous studies have suggested that the GPI-anchored anti-gp120 scFv X5 can interfere with HIV-1 Env glycoprotein processing and viral infectivity^48^. Herein, we exploited the mechanism underlying the potent inhibitory activity of GPI-m36.4. To exclude potential effects caused by the lentiviral vector, we further cloned the GPI-m36.4 and GPI-FluIgG03 fusion genes into the plasmid expression vector pcDNA3.1 and cotransfected them with an HIV-1 provirus clone (NL4-3 or THRO.c/2626) into HEK293T cells. As detected by FACS analysis, the two fusion genes were efficiently expressed on the surface of transfected cells (Fig. 7A). To determine the effects of the GPI-anchored antibodies on the release of HIV-1 virions, an enzyme-linked immunosorbent assay (ELISA) was used to quantify the HIV-1 P24 antigen in the cell culture supernatants. Cotransfection with GPI-m36.4 or GPI-FluIgG03 significantly reduced the production of the P24 antigen (Fig. 7B), indicating that virion release from the transfected cells was inhibited. Given that GPI-FluIgG03 also exhibited inhibitory activity, the expression of a membrane-anchoring scFv via GPI in lipid rafts might interfere with viral budding in a nonspecific manner, which was previously observed^48^. To test this hypothesis, we further constructed three GPI-anchored control antibodies targeting the spike protein of SARS-CoV-2, including one scFv (CB6) and two domain antibodies (5F8 and H11-H4)^49–51^. Similarly, the pcDNA3.1-based constructs produced efficient expression of GPI-CB6, GPI-5F8, and GPI-H11-H4 on HIV-1-producing cells (Supplementary Fig. S6), while GPI-CB6 interfered with the production of progeny HIV-1 virions (NL4-3 or THRO.c/2626); neither GPI-5F8 nor GPI-H11-H4 had a similar effect (Supplementary Fig. S7). These results imply that the larger size of a membrane-tethered scFv may cause steric hindrance that limits the budding of progeny viruses, whereas GPI-anchored nanobodies do not affect viral budding significantly, which should be characterized in more detail.

Next, we assessed the infectivity of HIV-1 virions released from cells expressing GPI-anchored antibodies. Cell culture supernatants containing equal amounts of the P24 antigen were used to infect TZM-bl cells, and the relative level of infection was determined by quantifying luciferase activity in cell lysates. Compared to the infectivity of HIV-1 strains produced from cells cotransfected with an empty vector (negative control), the infectivity of both HIV-1 NL4-3 and THRO.c/2626 produced from cells expressing GPI-m36.4 but not GPI-FluIgG03 was dramatically decreased (Fig. 7C).

**Fig. 8:**
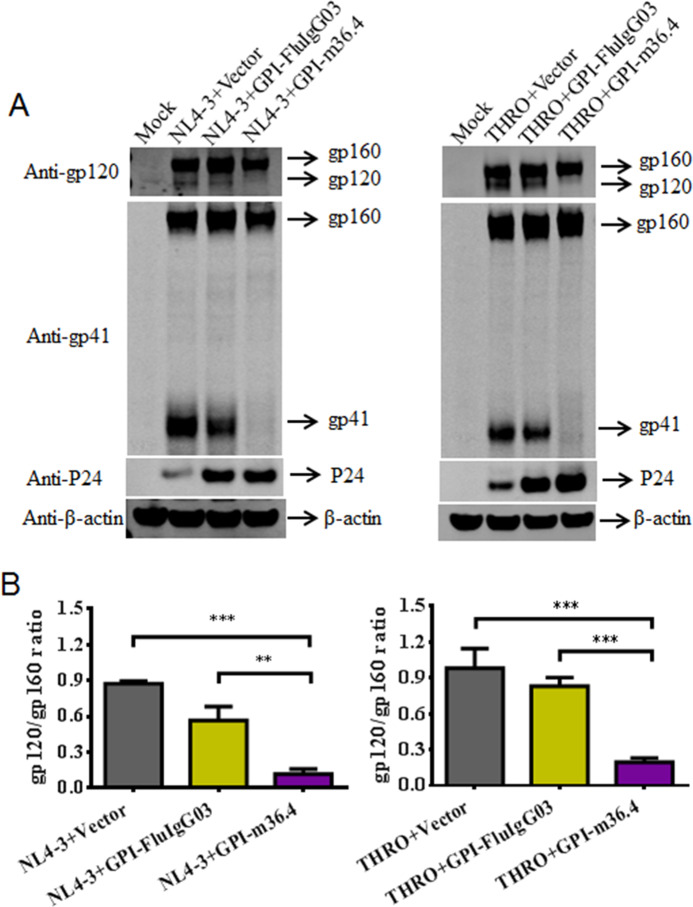
Effects of GPI-anchored antibodies on HIV-1 Env processing. **A** The expression levels of gp160, gp120, gp41, P24, and β-actin in HEK293T cells coexpressing GPI-m36.4 or GPI-FluIgG03 and NL4-3 (left panel) or THRO.c/2626 (right panel) were detected by Western blotting. For clarity, images were spliced and grouped, and the original images are provided in the Supplementary materials. **B** The ratio of gp120 to gp160 in cell lysates was determined by quantifying the corresponding band intensities with ImageJ. The data shown were derived from three independent experiments, and error bars indicate standard deviations. Statistical comparisons were conducted by ANOVA (**P < 0.01; ***P < 0.001)

To understand how GPI-m36.4 can dramatically impair the infectivity of progeny HIV-1 virions, we further characterized its impacts on the expression and processing profiles of Env glycoproteins when coexpressed with HIV-1 in HEK293T cells. As detected with a rabbit anti-gp120 polyclonal antibody in a Western blot assay, the amounts of both NL4-3 gp120 and THRO.c/2626 gp120 were sharply reduced in GPI-m36.4-cotransfected cells (Fig. 8A). Consistently, the ratio of gp120/gp160 was significantly reduced by GPI-m36.4 relative to the empty vector control and GPI-FluIgG03 (Fig. 8B). As detected with the human anti-gp41 monoclonal antibody 10E8, the amounts of NL4-3 gp41 and THRO.c/2626 gp41 were also sharply decreased upon cotransfection with GPI-m36.4. In contrast, the cotransduction of GPI-m36.4 had no obvious effect on the expression of the P24 antigen, which was released into the cell culture medium at high levels when a control vector was cotransfected. Taken together, these data suggest that GPI-m36.4 can interfere with Env processing in HIV-producing cells, which may critically determine viral infectivity.

## Discussion

In this study, we continued to explore novel strategies to effectively create HIV-resistant cells. As shown above, we finely characterized the single-domain antibody m36.4 for its potential as a membrane-bound viral entry inhibitor when attached via a GPI anchor. By applying a self-inactivating lentiviral vector, GPI-anchored m36.4 could be efficiently expressed on the plasma membrane of transduced TZM-bl cells and primarily directed to lipid raft sites without interfering with the expression of the cell receptor CD4 or coreceptors CCR5 and CXCR4. Transduced target cells displayed resistance to divergent cell-free HIV-1 infections, viral Env-mediated cell-cell membrane fusion and cell-associated virion-mediated cell-cell viral transmission. Moreover, we demonstrated that GPI-m36.4 could efficiently render human CD4^+^ T cells (CEMss-CCR5) nonpermissive to both CCR5- and CXCR4-tropic HIV-1 isolates and produced a robust survival advantage in transduced cells compared to unmodified cells during HIV-1 infection. Regarding its mechanism of action, GPI-m36.4 was also shown to impair HIV-1 Env processing and viral infectivity. In summary, our studies identified m36.4 as a viable inhibitor that can be used to generate HIV-resistant target cells, not only blocking infection at the entry step but also interfering with viral genesis.

Despite considerable progress, it is still disappointing that allogeneic transplantation of CCR5 gene-edited HSCs has achieved only minor therapeutic efficacy in patients^15,16,52^. In addition to the potential of off-target CCR5 editing, additional concerns increasingly arise over the deleterious effects of the CCR5Δ32 mutation. First, it renders cells resistant to only CCR5-tropic, not CXCR4-tropic, HIV-1 strains; second, a shift in HIV-1 tropism to CXCR4 usage would occur when CCR5 is disrupted^53^; and third, an increased susceptibility to some virus infections has been observed^54–56^. Compared to the deletion or disruption of host cell genes, we generally consider knocking in antiviral genes, especially those encoding HIV-1 entry inhibitors, to be a safer and more efficient way to modify cells for resistance. However, it should be realized that very few studies have focused on developing genetically virus-resistant cells with anti-HIV neutralizing antibodies, although emerging studies have already shown that bNAbs possess promising potential as soluble antibody drugs in clinical trials^6–9^. In view of this, we think that it is critical to characterize bNAbs for generating HIV-resistant cells. Hence, we concluded that GPI-m36.4 has high potential to be further developed as a novel HIV-1 gene therapy that can be used alone or in combination.

By applying a similar GPI-based anchoring method, Zhou and coworkers previously characterized several human or llama anti-HIV antibodies and a fusion inhibitor peptide (C34) as membrane-bound inhibitors^43,44,57–59^. Among several GPI-anchored scFvs, the authors found that the first-generation antibody X5, which also targets a gp120 CD4i epitope, could endow cells with relatively strong resistance when tested against multiple clades of HIV-1 strains^43^. Interestingly, it was also found that the GPI-anchored variable region (VHH) of the heavy chain-only llama antibody JM4 but not that of JM2 was able to render target cells resistant to both cell-free and cell-associated HIV-1 isolates^57^. Previous studies have demonstrated that JM4 binds to a gp120 site that overlaps with the CD4i epitope and neutralizes HIV-1 strains from subtypes A, B, C, A/E, and G in a CD4-dependent manner, whereas JM2 binds to a CD4bs epitope and neutralizes HIV-1 strains from subtypes B, C, and G^60,61^. These studies imply that epitope specificity critically determines the functionality of GPI-anchored anti-HIV antibodies in distinct formats. In this study, we identified CD4i epitope-specific m36.4 as a new GPI-anchored inhibitor that can efficiently modify HIV-resistant cells. Overall, we realized that antibodies targeting the gp120 CD4i epitope would confer broad and potent inhibitory activity against HIV-1 infection when genetically anchored to the cell membrane through a GPI attachment signal. Recently, Misra et al. conducted characterization of the mechanism of action of the GPI-anchored scFvs X5 and P16, revealing that GPI-scFvs could inhibit the processing and function of HIV-1 Env glycoproteins to restrict the production and infectivity of newly synthesized HIV-1 particles^48^. The authors proposed that “anti-Env GPI-scFvs therefore appear to be unique anti-HIV molecules as they derive their potent inhibitory activity by interfering with both early (receptor binding/entry) and late (Env processing and incorporation into virions) stages of the HIV life cycle”^48^. Consistently, we found that GPI-m36.4 could dramatically alter HIV-1 Env processing in cotransfected cells and that the progeny viruses largely lost their infectivity.

As a potential genetic intervention, GPI-X5 (scFv) was evaluated in a humanized mouse model^62^. The study found that GPI-X5-transduced primary CD4+ T cells were selected in the peripheral blood and lymphoid tissues upon HIV-1 infection and that transduced CD4+ T cells, after being cotransfused with HIV-infected cells, significantly reduced viral loads and viral RNA copy numbers^62^. As a new-generation anti-HIV bNAb, m36.4 was previously reported to significantly improve neutralizing breath and potency^35–37^. In our future studies, we would like to evaluate GPI-m36.4 for its antiviral efficacy in primary CD4+ T cells from healthy donors or HIV-1-infected individuals as well as its in vivo anti-HIV activity in a humanized mouse or nonhuman primate model. Ideally, GPI-m36.4 will be tested in combination with other gene therapy approaches, such as administration of the GPI-anchored fusion inhibitor peptide 2P23^38^. To advance toward a more efficient HIV-1 gene therapy approach for potential clinical studies, we are in the process of developing GPI-anchored inhibitors that possess multiple functionalities. Considering that any genetic manipulation has the potential to cause cancer in modified cells, the side effects of GPI-anchored inhibitors should be carefully characterized in the preclinical stage.

## Materials and methods

### Plasmids and cells

The following reagents were obtained through the AIDS Reagent Program, Division of AIDS, NIAID, NIH: TZM-bl indicator cells, which stably express large amounts of CD4 and CCR5 and endogenously express CXCR4 (from John C. Kappes and Xiaoyun Wu); a panel of molecular clones for generating replication-competent HIV-1 isolates, including NL4-3 (from Malcolm Martin), LAI.2 (from Keith Peden), SG3.1 (from Sajal Ghosh, Beatrice Hahn, and George Shaw), JRCSF (from Irvin S. Y. Chen and Yoshio Koyanagi), 89.6 (from Ronald G. Collman), THRO.c/2626, CH077.t/2627 and RHPA.c/2635 (all from John Kappes and Dr. Christina Ochsenbauer); plasmids encoding the “global panel” HIV-1 Envs as reference strains of subtypes A, B, C, G, A/C, A/E, and B/C that represent the global AIDS epidemic (from David Montefiori). The plasmid encoding DSP_1-7_ and 293FT cells stably expressing CXCR4/CCR5/DSP_8–11_ were kindly gifted by Zene Matsuda at the Institute of Medical Science of the University of Tokyo (Tokyo, Japan). The human CD4^+^ T cell line CEM-SS expressing CCR5 (CEMss-CCR5) was kindly gifted by Paul Zhou at the Institute Pasteur of Shanghai, Chinese Academy of Sciences, China. HEK293T cells were purchased from the American Type Culture Collection (ATCC) (Rockville, MD, USA).

### Generation of recombinant lentiviruses carrying transgenes

Recombinant lentiviral vectors expressing GPI-anchored transgenes were constructed as described previously^38,43^. A fusion gene sequentially encoding m36.4 or the scFv of FluIgG03, a His tag, and a GPI attachment signal (C-terminal 34 amino acid residues of DAF) was designed, synthesized (Genwiz, China) and then ligated into a self-inactivating lentiviral transfer vector carrying an hPGK promoter (pRRLsin.PPT.hPGK.WPRE) after restriction digestion with BamHI and SalI endonucleases. A fusion gene encoding GFP and GPI-m36.4 or FluIgG03 linked via an internal 2A peptide signal was ligated between the BamHI and SalI sites of a self-inactivating lentiviral transfer vector with an hEF1α promoter (pRRLsin-18.PPT.hEF1α.2A.GFP.WPRE). Recombinant lentiviruses expressing fusion genes were packaged as described previously^38^. In brief, 1.5 × 10^7^ HEK293T cells were seeded in P-150 dishes in 25 ml of complete DMEM and cultured overnight. By using a linear polyethyleneimine (PEI) transfection reagent, the cells were cotransfected with 50 μg of lentiviral transfer vector encoding GPI-anchored m36.4 or scFv, 18.75 μg of packaging construct delta8.9 encoding Gag/Pol/Rev, and 7.5 μg of plasmid encoding vesicular stomatitis virus G envelope (VSV-G). At 20 h posttransfection, the culture medium was replaced with fresh complete DMEM containing 10% FBS. After culturing for an additional 24 h, the virus-containing supernatants were harvested and centrifuged at 4000 rpm and 4 °C for 15 min, followed by filtration through a 0.45-mm filter; the supernatants were then concentrated by ultracentrifugation. The precipitated pellets were resuspended in RPMI 1640 medium with 25 mM HEPES and stored in aliquots in a −80 °C freezer. Lentivirus titers were determined with HEK293T cells according to a protocol described previously^38^. Briefly, lentiviruses were diluted and used to infect HEK293T cells, the expression of the His tag or GFP was monitored by flow cytometry (FACSCanto II; Becton, Dickinson, Mountain View, CA), and the titers are expressed as transducing units (TU) per milliliter.

### Generation of stable cell lines expressing GPI-anchored m36.4 or FluIgG03

Target cells stably expressing GPI-m36.4 or GPI-FluIgG03 (scFv) were generated as described previously^38,43^. Briefly, 5 × 10^4^ TZM-bl, 293FT, or CEMss-CCR5 cells/well were seeded in a 24-well plate and incubated overnight. A total of 2 × 10^6^ TU of recombinant lentiviruses was added to the cells and supplemented with 8 μg/ml polybrene (Sigma). After incubation for 24 h, the transduced cells were extensively washed and cultured in complete DMEM. TZM-bl and 293FT cells expressing the transgenes were sorted and collected using a mouse anti-His tag antibody (Invitrogen Life Technologies) and phycoerythrin (PE)-conjugated goat anti-mouse IgG antibody (eBioscience), and the CEMss-CCR5 cells expressing the transgenes were sorted by GFP expression.

### Flow cytometry analysis (FACS)

To analyze the cell-surface expression of GPI-m36.4 or GPI-FluIgG03, transduced TZM-bl, 293FT, or CEMss-CCR5 cells were sequentially stained with a mouse anti-His tag antibody and a PE-conjugated goat anti-mouse IgG antibody or Alexa Fluor 647-conjugated goat anti-mouse IgG antibody (Invitrogen Life Technologies) for 60 min at 4 °C. The stained cells were then washed twice with FACS buffer (phosphate-buffered saline [PBS] solution with 0.5% bovine serum albumin [BSA] and 2 mM EDTA) and resuspended in 0.2 ml of FACS buffer containing 4% formaldehyde. FACS analysis was conducted with a FACSCanto II instrument. To examine whether the expression of transgenes was truly linked with a GPI anchor, transduced TZM-bl cells were first treated with 5 U/ml PI-PLC (Invitrogen Life Technologies) in 0.5 ml of 1× PBS, rocked for 30 min at 4 °C, and then washed two times with FACS buffer to remove the remaining PI-PLC. Subsequently, the cells were stained with antibodies and analyzed by FACS analysis as described above. To determine whether GPI-anchored m36.4 or FluIgG03 scFv affected the expression of CD4, CCR5, and CXCR4, transduced cells were stained with a PE-conjugated anti-human CD4, CD195, or CD184 antibody or an allophycocyanin-labeled anti-human CD4, CD195 or CD184 antibody (BD Bioscience) for 30 min at 4 °C, washed twice with FACS buffer, and then fixed with 4% formaldehyde for FACS analysis.

### Confocal analysis

Colocalization of GPI-m36.4 or GPI-FluIgG03 with a lipid raft marker (GM1) was evaluated as described previously^38,43^. In brief, transduced TZM-bl cells were seeded (8000 cells/well) in a 35-mm glass dish with a 14-mm bottom well (Cellvis) and incubated for 2 days at 37 °C in 5% CO_2_. After two washes with PBS, the cells were fixed with 4% formaldehyde in PBS containing 1% BSA for 15 min and blocked with blocking buffer (5% goat serum in PBS containing 1% BSA) for 1 h. Then, the cells were sequentially stained with a mouse anti-His tag antibody, Alexa Fluor 488-conjugated goat anti-mouse IgG antibody, and Alexa Fluor 555-conjugated CtxB (Invitrogen Life Technologies). After three washes with PBS, the cells were further stained with DAPI in permeabilization buffer (blocking buffer plus 0.5% saponin) for 7 min. Images were captured with a laser confocal microscope (Leica Microsystems, Wetzlar, Germany).

### Real-time quantitative PCR analysis

To measure transgene-carrying lentivirus vector copy numbers (VCNs), genomic DNA from transduced cells was extracted using the QIAGEN DNeasy Blood & Tissue Kit according to the manufacturer’s instructions. Genomic DNA was used to detect the m36.4 transgene by using qPCR assays. Primer and probe sequences for this assay were as follows: 5′- TGGATCGGCGAGATTAACG-3′ (forward), 5′-TGCTGATGGTCACTCTGCTTTT-3′ (reverse), and 5′- CAGCGGCAATACCATCTACAACCCCA-3′ (probe in the 5′-FAM/3′-BHQ1 format). The primers and probe were synthesized by Tsingke Biological Technology (Beijing, China). Monoplex PCRs were set up in duplicate in 20-µl reaction volumes using Applied Biosystems TagMan Fast Advanced Master Mix in a 96-well plate on the Bio-Rad CFX96 real-time PCR platform. The thermocycling conditions were UNG incubation at 50 °C for 2 min, inactivation of reverse transcriptase at 95 °C for 2 min, and 40 cycles of PCR amplification (denaturing at 95 °C for 3 seconds, and annealing/extension at 60 °C for 30 s). The copy number of the m36.4 transgene was determined from the plasmid standard curve and normalized to the cell number.

### Inhibitory effects of GPI-anchored antibodies on replicative HIV-1 isolates

A panel of eight replication-competent HIV-1 isolates including NL4-3, LAI.2, JRCSF, 89.6, THRO.c/2626, CH077.t/2627, RHPA.c/2635, and MJ4 was generated by transfecting HEK293T cells (6 × 10^6^ cells in a P-100 mm dish) with 24 μg of plasmid encoding the molecular clone using a linear PEI transfection reagent. The virus-containing supernatants were harvested at 48 h posttransfection and stored in aliquots in a −80 °C freezer. The titers of the viruses were determined with a 50% tissue culture infectious dose (TCID_50_) assay performed with TZM-bl cells. To measure the inhibitory activity of GPI-m36.4 or GPI-FluIgG03 in transduced TZM-bl cells, a virus at 200 TCID_50_ was added to cells (1 × 10^4^/well) and incubated for 2 days at 37 °C in 5% CO_2_. Then, the cells were lysed with lysis buffer, and luciferase activity was quantitated with a BrightGlo luciferase assay by a luminescence counter (Promega). To measure the inhibitory activity of GPI-m36.4 in transduced CEMss-CCR5 cells, a virus at 1000 TCID_50_ was added to cells (1 × 10^6^/well) and incubated overnight at 37 °C in 5% CO_2_. The infected cells were extensively washed with Hanks’ balanced salt solution (HBSS) and then cultured in complete DMEM. Intracellular HIV-1 P24 expression in the infected cells was detected over time using a PE-conjugated anti-P24 Gag antibody (clone KC57, Beckman Coulter, Brea, CA, USA) and analyzed along with GFP expression by FACS analysis.

### Inhibitory effects of GPI-anchored antibodies on HIV-1 pseudoviruses

A single-cycle infection assay was carried out as described previously^38^. Briefly, the Env-pseudotyped viruses in the “global panel” of HIV-1 isolates and VSV-G were generated by transfection of HEK293T cells with a pSG3Δenv backbone plasmid and an Env-encoding plasmid using a linear PEI transfection reagent. The pseudovirus-containing culture supernatants were harvested at 48 h posttransfection and stored in aliquots in a −80 °C freezer. The titers of the pseudoviruses were determined with a TCID_50_ assay with TZM-bl cells. To measure the inhibitory activity of GPI-m36.4 or GPI-FluIgG03 in transduced TZM-bl cells, 200 TCID_50_ of virus was added to the cells (1 × 10^4^/well) and incubated at 37 °C for 48 h. The cells were lysed and quantitated for luciferase expression as described above.

### Inhibitory effects of GPI-anchored antibodies on HIV-1 Env-mediated cell-cell fusion

A dual-split-protein (DSP)-based cell–cell fusion assay was described previously^31^. To measure the inhibitory activity of GPI-m36.4 or GPI-FluIgG03, 293FT cells stably expressing CCR5/CXCR4/DSP_8–11_ (referred to as 293FT_Target_ cells) were first transduced with a lentiviral vector encoding GPI-m36.4 or GPI-FluIgG03. HEK293T cells (referred to as effector cells) were seeded in a 96-well plate (1.5 × 10^4^/well), incubated at 37 °C with 5% CO_2_ overnight, cotransfected with a DSP_1-7_-expressing plasmid and an Env-expressing plasmid and incubated at 37 °C for 24 h. GPI-m36.4-, GPI-FluIgG03, or mock-transduced 293FT_Target_ cells were resuspended in prewarmed culture medium, added to EnduRen live cell substrate (Promega) and then incubated at 37 °C for 30 min. The target cells (2.5 × 10^4^/well) were added to the effector cells and spun down to maximize cell-cell contact. After coculturing for 6 h, cell–cell fusion activity was quantitated by measuring the production of the luciferase reporter as described above.

### Inhibitory effects of GPI-anchored antibodies on cell–cell HIV-1 transmission

A cell-cell viral transmission assay was conducted according to a protocol described previously^38^ based on the fact that infection of TZM-bl cells by many R5-tropic viruses as cell-free virions depends on polycationic supplements but that cell–cell transmission does not depend on these supplements. TZM-bl cells were transduced with GPI-m36.4 or GPI-FluIgG03 as a target, and CEMss-CCR5 cells were infected with a CCR5-tropic virus (RHPA.c/2635, THRO.c/2626, or MJ4) as a donor. After donor cells and target cells were cocultured for 36 h, infection of TZM-bl cells was evaluated by quantifying the production of the luciferase reporter as described above.

### Inhibitory effects of GPI-anchored antibodies on HIV-1 production and infectivity

To determine the effects of GPI-m36.4 on HIV-1 production and infectivity, the expression cassettes of GPI-m36.4 and controls (GPI-FluIgG03, GPI-CB6, GPI-5F8, and GPI-H11-H4) were subcloned into the plasmid expression vector pcDNA3.1. HEK293T cells were cotransfected with 0.8 μg of pcDNA3.1 vector and 0.8 μg of proviral clone of HIV-1 NL4-3 or transmitter/founder HIV-1 strain THRO.c/2626 by using a linear PEI transfection reagent. After 12 h, the cell culture supernatants were replaced with fresh DMEM and incubated for an additional 36 h. The virus-containing supernatants were harvested and centrifuged at 4000 rpm and 4 °C for 15 min, followed by filtration with a 0.45-mm filter. The cotransfected cells were collected to analyze the cell-surface expression of GPI-anchored antibodies by FACS with an anti-His antibody as described above. To examine virions released from the cotransfected cells, the level of the HIV-1 P24 antigen in the culture supernatants was quantified by ELISA. To examine the infectivity of the progeny virions, the collected supernatants containing 0.25 ng of P24 antigen were added to TZM-bl cells (1 × 10^4^/well) and incubated for 2 days at 37 °C in 5% CO_2_. Then, the cells were lysed and quantitated for luciferase expression as described above.

### Inhibitory effects of GPI-anchored antibodies on HIV-1 Env processing

To determine the effect of GPI-m36.4 on HIV-1 Env processing, HEK293T cells expressing a transgene and an HIV-1 proviral clone (NL4-3 or THRO.c/2626) were used to analyze the expression and processing of viral gp160 by Western blotting. In brief, cells were lysed in ice-cold RIPA buffer (Invitrogen) containing protease inhibitors (Roche) to isolate proteins. The cell proteins were separated by SDS-PAGE and transferred to a nitrocellulose membrane, which was then blocked with a 5% nonfat dry milk solution in TBS-Tween 20 at room temperature for 1 h. The membrane was incubated overnight at 4 °C with a rabbit anti-gp120 polyclonal antibody (SinoBiological, Beijing, China), the human anti-gp41 monoclonal antibody 10E8, a mouse anti-P24 antibody (Abcam), or a mouse anti-β actin antibody (Sigma). After washing three times with TBS-Tween 20, the membrane was incubated with IRDye 680LT goat anti-rabbit IgG, IRDye 800CW goat anti-human IgG, or IRDye 680RD goat anti-mouse IgG for 2 h at room temperature. Images were acquired with an Odyssey infrared imaging system (LI-COR Biosciences, Lincoln, NE, USA). The images were spliced and grouped for clarity.

### Statistical analysis

GraphPad Prism 6 software was applied to statistically analyze data. One-way analysis of variance with Tukey’s multiple comparisons was used to identify significant differences among three or more groups. A *P* value < 0.05 was considered significant.

## Supplementary information

Figure S1

Figure S2

Figure S3

Figure S4

Figure S5

Figure S6

Figure S7

Supplementary Information

## Data Availability

The datasets generated for this study are available on request from the corresponding author.
