# Supplementary figures and images for: Generation of HIV-resistant cells with a single-domain antibody: implications for HIV-1 gene therapy
Source: Cell Mol Immunol. 2021 Jan 18;18(3):660–74. doi: 10.1038/s41423-020-00627-y (PMC7812570; doi:10.1038/s41423-020-00627-y)

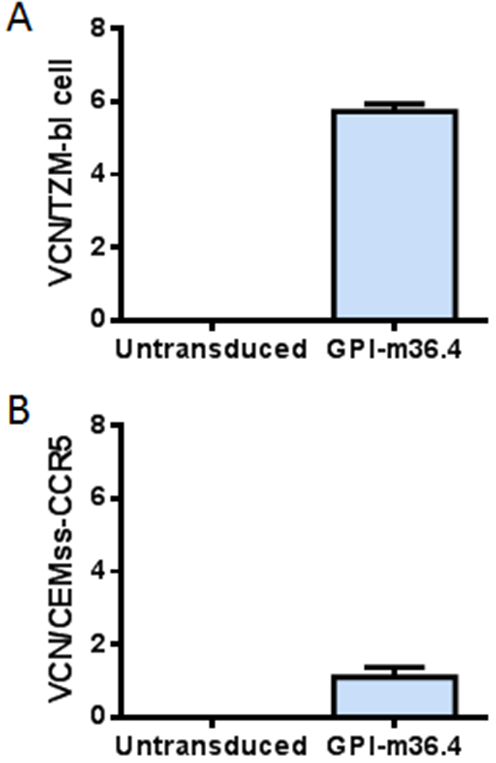

Supplement: Supplementary file 1 — Figure S1 [file 41423_2020_627_MOESM1_ESM.tif]

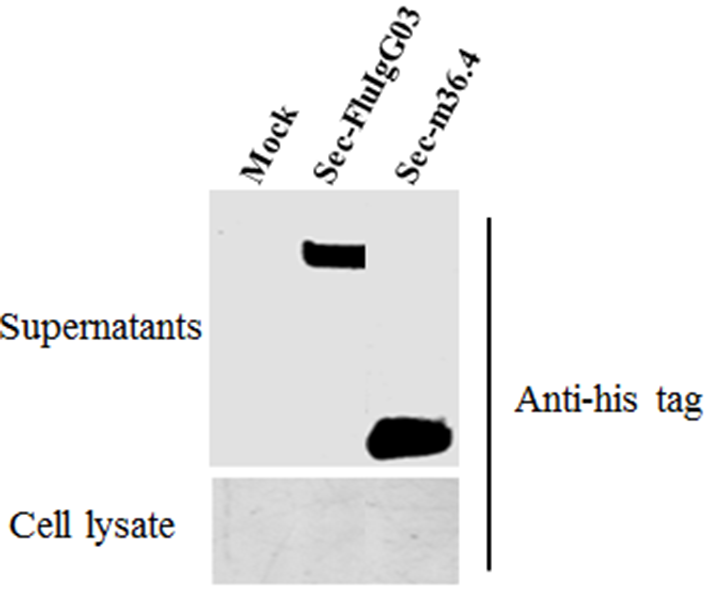

Supplement: Supplementary file 2 — Figure S2 [file 41423_2020_627_MOESM2_ESM.tif]

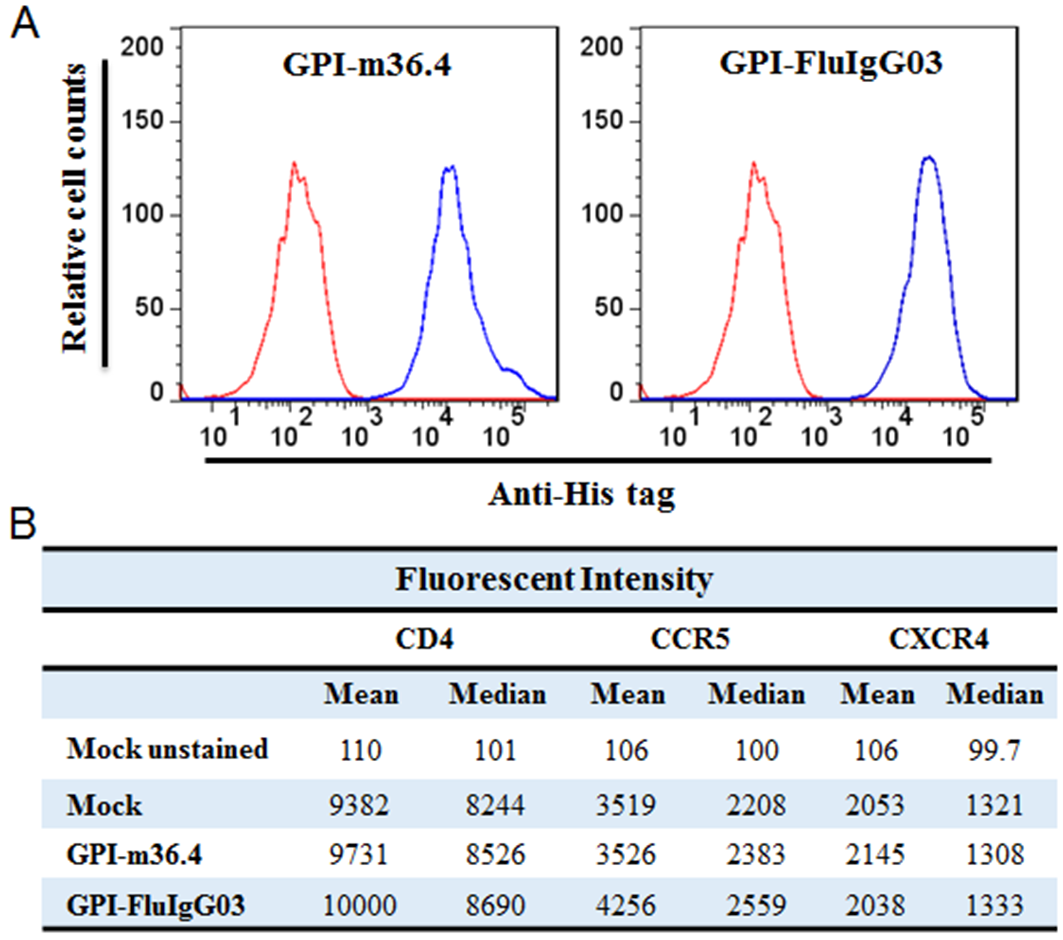

Supplement: Supplementary file 3 — Figure S3 [file 41423_2020_627_MOESM3_ESM.tif]

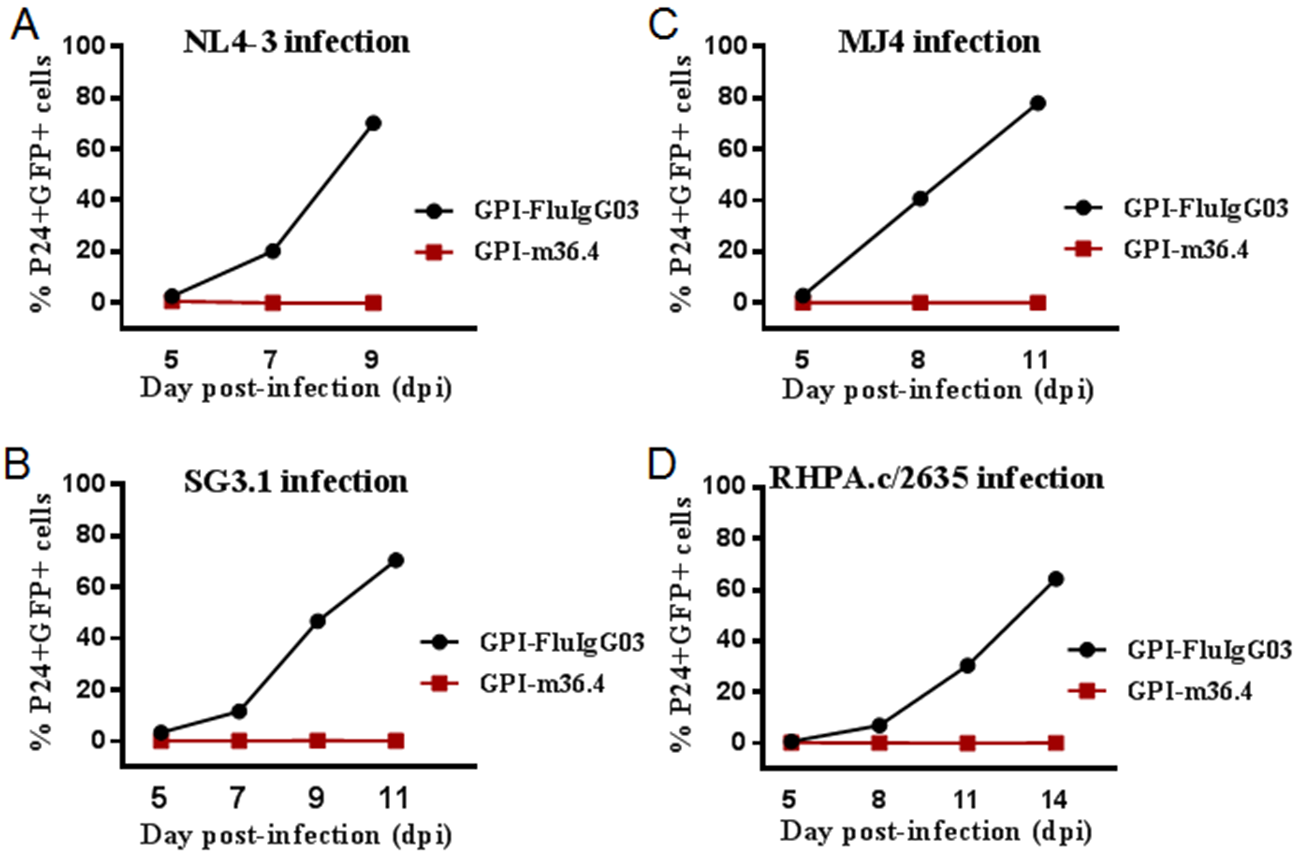

Supplement: Supplementary file 4 — Figure S4 [file 41423_2020_627_MOESM4_ESM.tif]

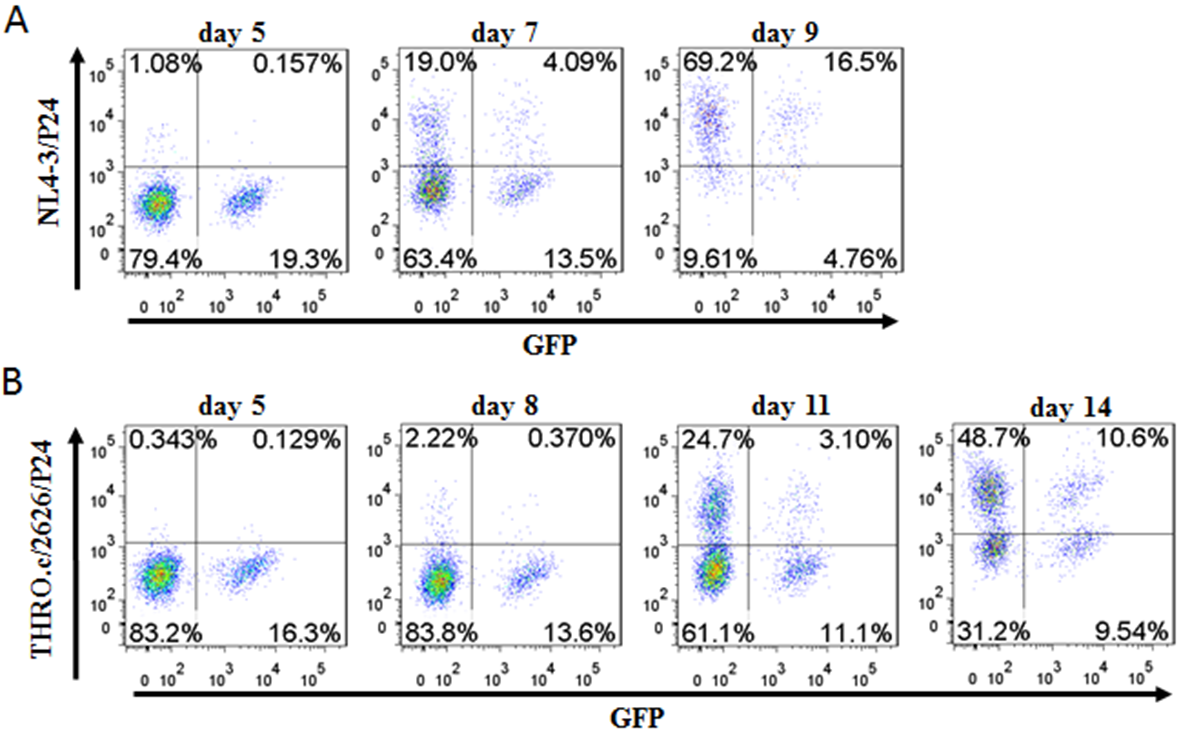

Supplement: Supplementary file 5 — Figure S5 [file 41423_2020_627_MOESM5_ESM.tif]

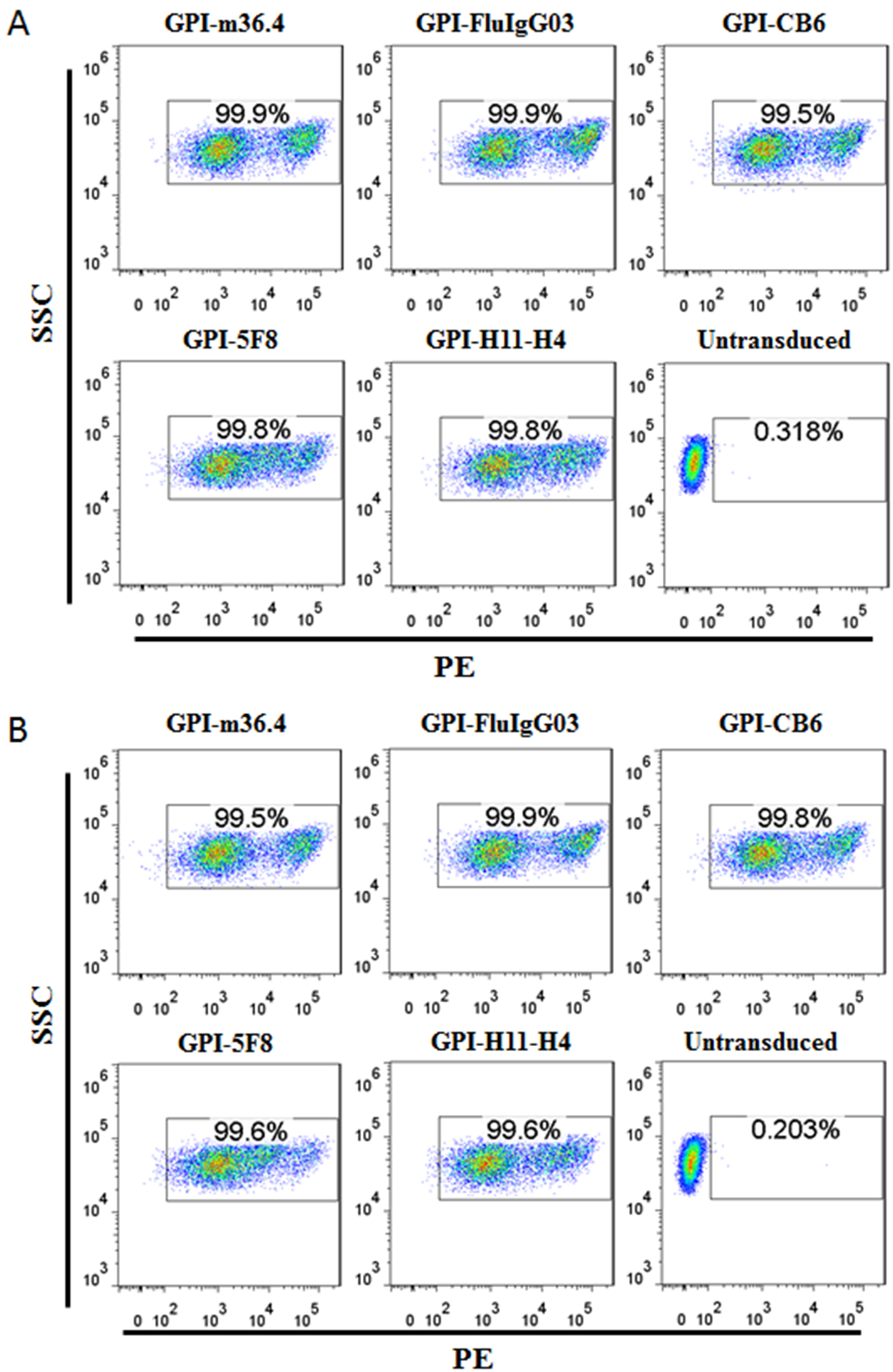

Supplement: Supplementary file 6 — Figure S6 [file 41423_2020_627_MOESM6_ESM.tif]

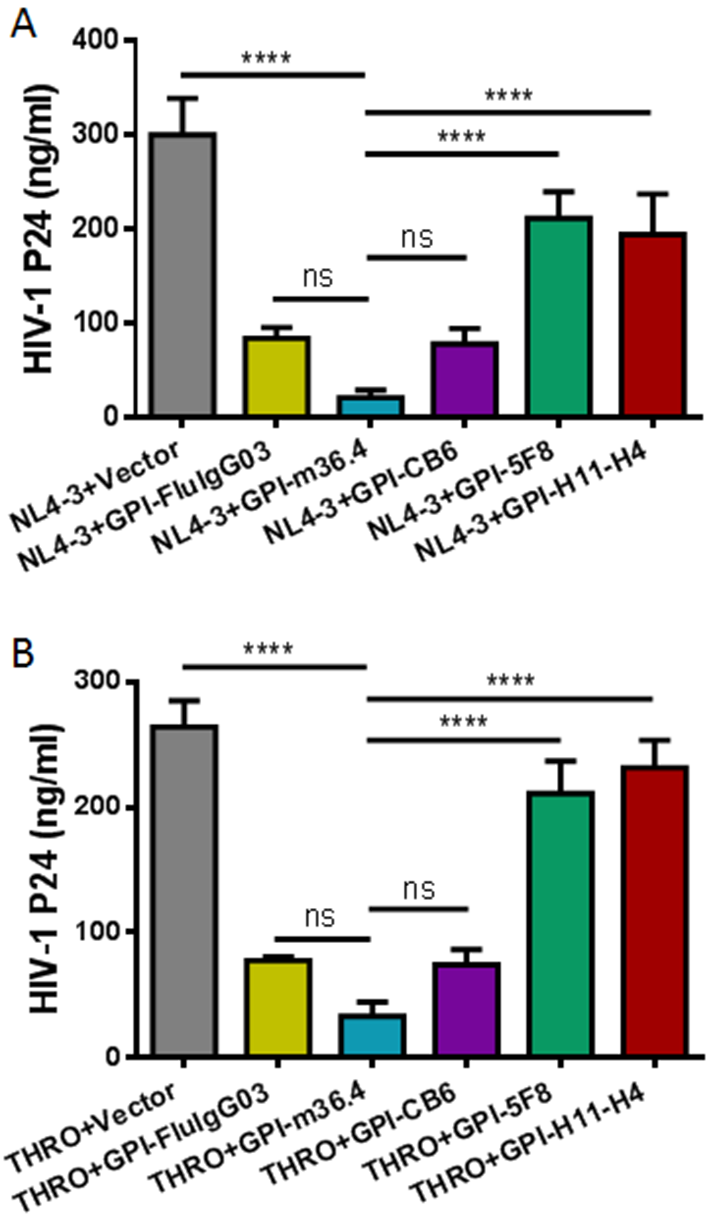

Supplement: Supplementary file 7 — Figure S7 [file 41423_2020_627_MOESM7_ESM.tif]
