# Supplementary material for: Generation of HIV-resistant cells with a single-domain antibody: implications for HIV-1 gene therapy
Source: Cell Mol Immunol. 2021 Jan 18;18(3):660–74. doi: 10.1038/s41423-020-00627-y (PMC7812570; doi:10.1038/s41423-020-00627-y)
Supplement: Supplementary file 8 — Supplementary Information [file 41423_2020_627_MOESM8_ESM.docx]

**Figure S1. Quantification of vector copy number (VCN) in transduced cells by real-time qPCR.**

(**A**) VCN of the lentiviral vector pRRLsin.PPT.hPGK.WPRE expressing GPI-m36.4 in transduced TZM-bl cells.

(**B**) VCN of the lentiviral vector pRRLsin-18.PPT.EF1α.WPRE expressing GPI-m36.4 in transduced CEMss-CCR5 cells.

The assay was performed with duplicate samples and repeated three times, and data are expressed as the means ± SD.

**Figure S2. Expression of secretory m36.4 and FluIgG03 in transduced TZM-bl cells.** Lentiviral vectors encoding secretory m36.4 (sec-m36.4) or FluIgG03 (sec-FluIgG03) with a His tag were constructed and transfected into TZM-bl cells. Secretory antibodies in highly concentrated cell culture supernatants and lysates were detected with a mouse anti-His tag antibody by Western blotting.

**Figure S3. Expression of GPI-anchored antibodies in transduced 293FT target cells expressing CCR5/CXCR4/DSP_8-11_ and effects of the antibodies on CD4, CCR5, and CXCR4.**

**(A)** The expression of GPI-m36.4 or GPI-FluIgG03 on the surface of transduced 293FT cells, which express CCR5/CXCR4/DSP_8-11_, was detected with a mouse anti-His tag antibody and analyzed by FACS analysis. The red histogram represents the relative cell count of mock-transduced cells, and the blue histogram represents the relative cell count of GPI-m36.4- or GPI-FluIgG03-transduced cells.

**(B)** Expression of CD4, CCR5 or CXCR4 on the surface of transduced cells was detected with a PE-conjugated anti-human CD4, CCR5 or CXCR4 antibody and analyzed by FACS analysis. The expression levels were assessed based on the fluorescence intensity.

**Figure S4. Inhibitory effects of GPI-anchored antibodies in transduced human CD4+ T cells on HIV-1 infection.**

CEMss-CCR5 cells transduced with GPI-m36.4/GFP or GPI-FluIgG03/GFP were infected with 1,000 TCID_50_ of indicated virus. Based on intracellular HIV-1 P24 Gag and GFP expression, the infection curves of NL4-3- **(A),** SG3.1- **(B)**, MJ4- **(C),** and RHPA.c/2635 **(D)**-infected cells are shown. Data from an experiment representative of three independent experiments are shown.

**Figure S5. Selective survival of CEMss-CCR5 cells expressing GPI-FluIgG03 in the context of HIV-1 infection.**

CEMss-CCR5 cells were transduced with GPI-FluIgG03/GFP and mixed with untransduced cells at a proportion of approximately 17% GFP-positive cells. The mixed population was challenged with 1,000 TCID_50_ of NL4-3 **(A)** or THRO.c/2626 **(B)**, and the proportion of transgene-expressing cells was monitored over time by flow cytometry.

**Figure S6. Cell-surface expression levels of GPI-anchored scFvs or domain antibodies.**

HKE293T cells were cotransfected with GPI-m36.4, GPI-FluIgG03, GPI-CB6, GPI-5F8, or GPI-H11-H4 and a proviral clone of HIV-1 NL4-3 (**A**) or THRO.c/2626 (**B**). The expression levels of GPI-anchored antibodies were detected by FACS analysis with an anti-His tag antibody. SSC, side scatter.

**Figure S7. Effect of GPI-anchored antibodies on the release of progeny HIV-1 virions.**

HEK293T cells were cotransfected with GPI-m36.4, GPI-FluIgG03, GPI-CB6, GPI-5F8, or GPI-H11-H4 and the HIV-1 provirus NL4-3 (**A**) or THRO.c/2626 (**B**). The amounts of the P24 antigen in cell culture supernatants were measured by ELISA. The data shown were derived from three independent experiments, and error bars indicate standard deviations. Statistical comparisons were conducted by ANOVA (*, *P* < 0.05; **, *P* < 0.01; ****, *P* < 0.0001; ns, not signiﬁcant).
